# Evaluation of the Use of TRIzol-Based Protein Extraction Approach for Gel-Based Proteomic Analysis of Dried Seafood Products and Chinese Tonic Foods

**DOI:** 10.3390/ijms19071998

**Published:** 2018-07-09

**Authors:** Kin-Ka Chan, Celia Sze-Nga Kwok, Eric Tung-Po Sze, Fred Wang-Fat Lee

**Affiliations:** School of Science and Technology, The Open University of Hong Kong, HKSAR, Hong Kong, China; kkchan@ouhk.edu.hk (K.-K.C.); snkwok@ouhk.edu.hk (C.S.-N.K.)

**Keywords:** protein extraction, TRIzol, two-dimensional gel electrophoresis

## Abstract

Although the emergence of gel-free approaches has greatly enhanced proteomic studies, two-dimensional gel electrophoresis (2-DE) remains one of the most widely used proteomic techniques for its high resolving power, relatively low cost, robustness, and high resolution. Preparation of high-quality protein samples remains the key in high-quality 2-DE for proteomic analysis. Samples with high endogenous levels of interfering molecules, such as salts, nucleic acids, lipids, and polysaccharides, would yield a low-quality 2-DE gel and hinder the analysis. Recently, a TRIzol-based protein extraction method has gained prominence and has attracted attention due to its promising performance in high-quality 2-DE. The authors evaluate the use of this approach for four valuable dried food products, namely two dried seafood products (abalone slices and whelk slices) and two traditional Chinese tonic foods (ganoderma and caterpillar fungus). The results indicate that 2-DE gels obtained through the TRIzol-based method are of high-quality and are comparable to those obtained through the trichloroacetic acid–acetone method in terms of spot number, spot intensity, and resolution. The TRIzol-based method is generally applicable to dried food samples and is simple and fast, which greatly streamlines the protein extraction procedure. Additionally, it enables the concurrent extraction and analysis of RNA, DNA, and protein from the same sample.

## 1. Introduction

Dried seafood products and traditional Chinese tonic foods are commonly found in Chinese markets. They are key ingredients in Chinese cooking and some are highly valued due to their nutritional and medicinal properties. The market price of the caterpillar fungus species *Ophiocordyceps sinensis* is approximately HK$50/g in China [1], for example. Moreover, the general selling price of dried abalone (sometimes sold in slices) ranges from a few thousand to over 10,000 Hong Kong dollars per catty (604.79 g) [2]. Therefore, highly valued genuine species are often adulterated with low-valued species that are considerably cheaper. The Hong Kong Customs and Excise Department, in 2010, discovered more than 100 catties of fake dried abalone slices in a series of raids on 31 dried seafood retail shops in Hong Kong [2], for instance. The fake dried abalone slices were all found to be dried whelk slices instead. Customers would have difficulty identifying the adulterants based on the physical appearance, colour, taste, and texture of the food products alone without experimental examination. Therefore, the development of testing technology is crucial for the authentication of these valuable dried food products. 

Proteomic technologies are powerful tools for the large-scale study of the proteins encoded by an organism [3]. These technologies provide a platform to determine biological mechanisms and discover protein biomarkers [4,5]. Gel-free approaches, such as multidimensional liquid chromatography and protein arrays, have been developed in recent years and have further enhanced the research tools available in proteomic analysis [6,7]. However, two-dimensional gel electrophoresis (2-DE) remains one of the most widely used protein separation techniques due to its high resolution and low cost [8]. Two-dimensional gel electrophoresis is a powerful technique that separates a mixture of proteins into individual protein spots according to their isoelectric point (pI) and molecular weight (MW) under defined conditions. The protein spots can be easy visualised on the gel, and they can be isolated and identified using matrix-assisted laser desorption/ionisation time-of-flight mass spectrometry (MALDI-TOF MS) with a peptide mass fingerprint (PMF) and de novo peptide sequencing approaches. Proteomics analysis has been widely used in food authentication studies and food technology research [9,10], and 2-DE has been used to analyse the proteome of traditional Chinese tonic foods such as ganoderma and caterpillar fungus [11,12]. Although the production of 2-DE gel includes many steps, obtaining a quality protein sample remains the most important precondition for high-quality 2-DE. Samples including compounds such as nucleic acids, polysaccharides, lipids, pigments, and salts can severely affect the isoelectric focusing (IEF) process (the first step of 2-DE), which leads to smearing and streaking in the 2-DE gel [13,14,15,16]. Thus, tedious and lengthy preparatory steps are required to remove these endogenous contaminants. 

The clean-up procedures are typically time consuming and laborious, possibly taking several days. The trichloroacetic acid (TCA)-acetone method is a conventional approach used to extract protein from samples with complex matrices, especially plant samples [17,18]. It is effective in the removal of interfering compounds, and it produces high-quality protein samples due to its effective precipitation of proteins. Although TRIzol reagent (a phenol-guanidine isothiocyanate solution) is typically employed to isolate RNA from cells and tissues, it has been widely applied to extract proteins for 2-DE. This TRIzol-based extraction method has gained prominence and attracted attention due to its promising performance in removing contaminants from protein samples and, thus, facilitating high-quality 2-DE. The TRIzol-based extraction method was first applied successfully on halophilic protein samples for the generation of 2-DE in 2006 [19]. This protein extraction method has subsequently been widely used in 2-DE for a wide range of samples, including rat spinal cord [20], human/animal cell lines [21,22,23], dinoflagellates [11,24,25,26,27,28], mites [29], plant tissue [30], clinical samples [31,32,33], marine animals [34,35,36], and reef corals [37] (Table 1). This TRIzol-based method has gained popularity for 2-DE because it offers several advantages [19,24]. Compared with other conventional methods, the TRIzol-based method is convenient and fast. Soluble protein samples can usually be obtained in less than 4 h. Additionally, it effectively removes interfering compounds and renders samples fully compatible with IEF. More importantly, DNA and RNA can be extracted together with the proteins from a single sample. Some studies have demonstrated that analysing all such macromolecules from a single specimen would enable the integration of transcriptomic and proteomic data and thus provide a more comprehensive view of the regulatory mechanisms [20,22,33]. However, few studies have investigated protein extraction from dried seafood and traditional Chinese tonic foods for 2-DE.

The authors aim to evaluate the effectiveness of the TRIzol-based protein extraction method for 2-DE for dried seafood and traditional Chinese tonic foods. Four valuable dried food products—two dried seafood products (abalone slices and whelk slices) and two traditional Chinese tonic foods (ganoderma and caterpillar fungus)—are used in the study. Gels produced though the TRIzol-based method are compared with those produced through the TCA-acetone method in terms of protein yield, spot number, spot intensity, and resolution. Moreover, TRIzol-based 2-DE profiles of abalone slices and whelk slices are compared and corresponding differentially expressed proteins are identified. The results are intended to provide a useful foundation for the development of new authentication methods for dried seafood and traditional Chinese tonic foods through gel-based proteomic analysis. 

## 2. Results and Discussion

### 2.1. TRIzol Protein Extractions

Protein extraction using lysis buffer solutions (typically 7 M urea, 2 M thiourea, 4% 3-[(3-cholamidopropyl)dimethylammonio]-1-propanesulfonate (CHAPS), 40 mM Tris, 65 mM dithiothreitol (DTT), and 0.2% Biolyte buffer) is preferable because the method is easy and straightforward. More importantly, high protein recovery and high protein yield can always be achieved [36]. The lysis buffer method is usually effective for samples with few interfering compounds, such as animal and cancer cell lines and serum samples [40,41]. However, samples with complex matrices require extensive clean-up procedures or protein extraction methods that remove contaminants with high efficiency. The quality of the two-dimensional gel electrophoresis (2-DE) obtained using the lysis buffer method from the dried food samples used in this study was poor. The 2-DE gel exhibited high background signals and excessive horizontal and vertical streaks with very few spots. The poor-quality gel was attributed to the endogenous interfering compounds contained in the dried food samples that strongly affected the focusing of the protein spots during the IEF step.

Compared with the TCA-acetone method, the TRIzol method is considerably simpler, more convenient, faster, and more cost effective [24]. Additionally, the simultaneous analysis of gene expressions and protein expressions from an identical sample is a unique advantage offered by the TRIzol method. Moreover, RNA originating from the same sample could be useful for the validation of differentially expressed proteins identified in a comparative 2-DE study, especially for precious samples (clinical and field samples) that are available only in limited amounts [42]. Although TRIzol extraction has been successfully applied in a wide range of samples, Knigge et al. highlighted some potential drawbacks associated with the TRIzol method [42]. Additionally, for unknown reasons, unsatisfactory 2-DE gels have been obtained for particular samples through TRIzol extraction [43,44]. However, these results could be improved by introducing modified washing steps into the TRIzol extraction process [19,23].

### 2.2. Protein Concentrations and Number of Spots

**Figure 1:**
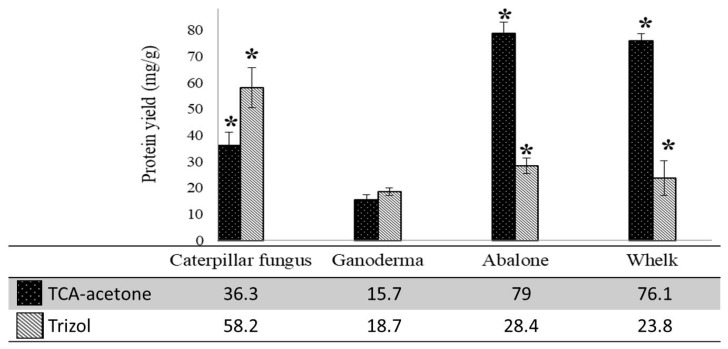
The comparisons of protein yields of caterpillar fungus, ganoderma, abalone, and whelk samples prepared by trichloroacetic acid (TCA)-acetone precipitation and TRIzol extraction methods. Results were the average of values from three independent experiments. * indicates significant difference (*p* ≤ 0.05) between two methods.

TRIzol and TCA-acetone extraction were applied to four dried food samples, and the protein yields were evaluated (Figure 1). The protein yields for the two dried traditional tonic foods (58.2 mg/g for caterpillar fungus and 18.7 mg/g for ganoderma) obtained through TRIzol extraction were higher than those obtained through TCA-acetone extraction (36.3 ± 4.8 mg/g for caterpillar fungus and 15.7 ± 1.7 mg/g for ganoderma). Notably, the opposite result was observed for the two dried seafood samples. The protein yields for the abalone (79 ± 3.9 mg/g) and whelk (76.1 ± 2.6 mg/g) samples obtained through TCA-acetone extraction were significantly (approximately threefold) higher than those obtained through TRIzol extraction (28.4 ± 3 mg/g for abalone and 23.8 ± 6.5 mg/g for whelk). Whether protein yield is a critical concern mainly depends on the availability and preciousness of the sample. Typically, 100–200 μg of total proteins is sufficient for silver staining 2-DE gel, and 1–2 mg of total proteins is sufficient for Coomassie Brilliant Blue staining. Therefore, the 23–28 mg of proteins extracted from the dried seafood samples through the TRIzol method was far more than required for the 2-DE analysis. The differences in protein yields might be attributed to different protein compositions in the two sample types. Some proteins might have higher solubilisation in the TRIzol extraction, whereas others might have higher solubilisation in the TCA-acetone method [33]. Additionally, some proteins might be lost through coprecipitation with ethanol, which is used for DNA precipitation in the TRIzol method [33]. However, the number of spots on the 2-DE gels for dried abalone and whelk produced through the TCA-acetone method were comparable to that of the TRIzol method. This indicates that protein loss might not be the case. 

**Figure 2:**
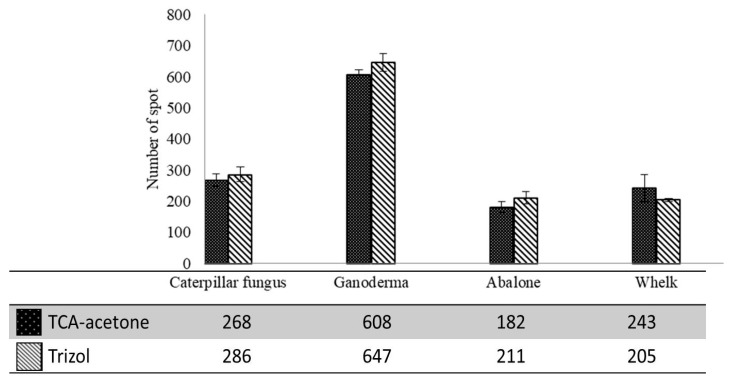
Total spot numbers on the 2-DE of caterpillar fungus, ganoderma, abalone, and whelk samples. Results were the average of values from three independent experiments.

The numbers of protein spots on the 2-DE gels produced though the TRIzol and TCA-acetone methods were determined using 2-DE analysis software Melanie VII (GeneBio, Genève, Switzerland) (Figure 2). The average number of spots on the 2-DE gels for caterpillar fungus, ganoderma, abalone, and whelk produced through the TCA-acetone method were 268 ± 20, 608 ± 15, 182 ± 17, and 243 ± 43, respectively. Similarly, the 2-DE gels obtained though the TRIzol method yielded an average of 286 ± 23, 647 ± 28, 211 ± 19, and 205 ± 5 spots for caterpillar fungus, ganoderma, abalone, and whelk, respectively. Unlike the results for protein yield, the numbers of protein spots on the 2-DE gels produced through the TRIzol and TCA-acetone methods were highly similar. The average numbers of common protein spots across the gels from both methods for caterpillar fungus, ganoderma, abalone, and whelk were around 154 ± 36, 498 ± 84, 137 ± 29 and 171 ± 41 respectively. Given that each protein extraction method has its own bias in extracting proteins; it is not surprising that a substantial amount of differing protein spots were found between the gels from the two methods. A study using TRIzol extraction demonstrated approximately 700 protein spots on the 2-DE gel of fresh abalone muscle [35]. However, only approximately 200 protein spots were obtained from the dried abalone sample in the current study (Figure 2). The large difference in the number of spots might be caused by the degradation of proteins during the drying process. This might also explain why the 2-DE gels of all the dried food products revealed only several hundred spots. 

### 2.3. Two-Dimensional Gel Electrophoresis (2-DE) Images

**Figure 3:**
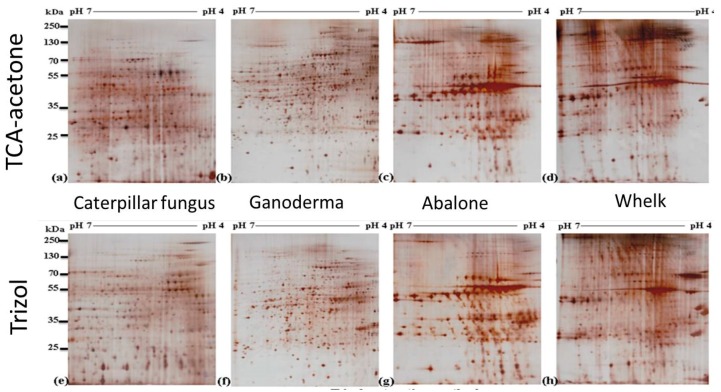
2-DE gels of total protein extracts (100 µg) of caterpillar fungus (**a**,**e**), ganoderma (**b**,**f**), abalone (**c**,**g**), and whelk (**d**,**h**) samples were loaded onto 18 cm immobilised pH gradient (IPG) strip with a pH range of 4–7 using TCA-acetone precipitation method (**a***–***d**) and Trizol extraction method (**e***–***h**). The silver staining method was used.

**Figure 4:**
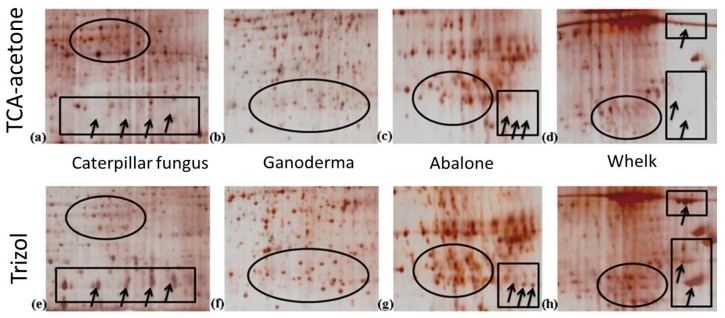
The enlargement of selected regions of 2-DE gels of caterpillar fungus (**a**,**e**), ganoderma (**b**,**f**), abalone (**c**,**g**), and whelk (**d**,**h**) samples. Circle areas in (**a**,**e**) indicated the background difference. Circle areas in (**b**–**d**) and (**f**–**h**) indicated the differences of intensity of spots. Square areas indicated the missing of protein spots. Arrows indicate the differentially expressed protein spots between the gels.

TCA-acetone protein extraction is a conventional method used for samples with complex matrices. It is usually effective for such “difficult” samples and produces high-quality 2-DE gel images with high resolution. The TCA-acetone method used in this study was effective for the four dried food samples (Figure 3). However, the qualities of the 2-DE gels of the samples obtained through TRIzol extraction were comparable to those obtained through the TCA-acetone method. Generally, the overall patterns of the gels produced through both methods were highly similar. Protein spots on the gels from both methods exhibited high resolution, were evenly distributed in the pH range of 4–7, and were mostly located between 25–80 kDa. Although some minor vertical streaks were observed in the gels of caterpillar fungus and whelk samples, the protein spots were focused, and no aberrant patterns were observed. The clarity and resolution of the 2-DE gels obtained through both methods were of sufficient quality to be used in proteomic studies. Nevertheless, for particular regions, high background signals were observed in the gels produced through the TCA-acetone method. High background signals were observed in the region located at a pH of 5–6 and MW of 25–55 kDa in the gels of caterpillar fungus, for example. However, a considerably clearer background was observed in the same region of the gel obtained through the TRIzol method (Figure 3). This is easily observable in the circled regions in enlarged areas of the gels shown in Figure 4a,e. This indicates that some interfering compounds were not completely removed during the TCA-acetone precipitation steps, and the contaminants, therefore, were coextracted with the proteins. To contrast, the intensities of some spots on the 2-DE gel prepared using TRIzol were considerably higher than those on the 2-DE gel prepared using TCA-acetone (Figure 4). The protein spots in the circled regions of Figure 4b–d were of lower intensity than those of Figure 4f–h, respectively, for example. The faint protein spots shown in the TCA-acetone-prepared 2-DE gel were clear in the TRIzol-prepared 2-DE gel. Additionally, presumably the clearer background of the gels prepared through the TRIzol method caused a clear increase in the spot numbers in these regions. Interestingly, the authors also found that some protein spots were missing and not observed in the gels prepared through the TCA-acetone method but appeared in the TRIzol-prepared gels (see spots in the rectangular regions in Figure 4). This could be attributed to the fact that each protein extraction method has a bias for extracting proteins. Due to the substantial diversity in sample types, no universal method is applicable for the efficient isolation of all proteins of interest. Generally, the chosen protein extraction method should efficiently remove contaminants and be simple, fast, and low cost. Having these advantages, the TRIzol protein extraction method is highly feasible and recommended for 2-DE proteomic analysis of dried seafood products and traditional Chinese tonic foods. 

### 2.4. Identification of Differentially Expressed Proteins Between Abalone Slices and Whelk Slices through Matrix-Assisted Laser Desorption/Ionisation Time-of-Flight Mass Spectrometry (MALDI-TOF/TOF MS)

**Figure 5:**
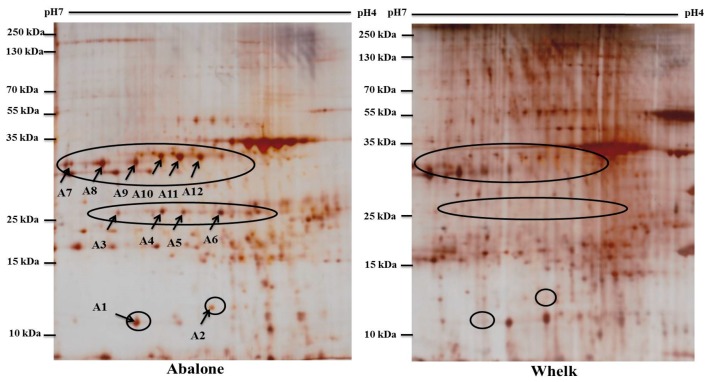
Representative 2-DE gels (prepared by TRIzol method) of whelk (**right image**) and abalone (**left image**) samples. Circled areas indicated the differentially expressed spots with a 2-fold difference between abalone and whelk samples. Arrows indicate the locations of protein spot A1 to A12.

**Figure 6:**
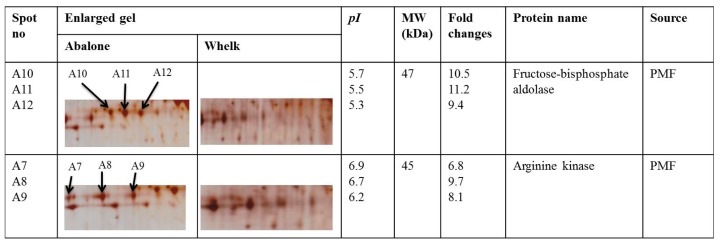
Differentially expressed protein spots identified successfully by matrix-assisted laser desorption/ionisation time-of-flight mass spectrometry (MALDI-TOF/TOF MS).

To demonstrate the feasibility of using TRIzol-prepared 2-DE for authentication analysis on these dried food products, comparative 2-DE analysis was conducted for the abalone and whelk samples (Figure 5). The general patterns of their 2-DE profiles were similar. Approximately 172 protein spots matched between the 2-DE gels of the abalone and whelk samples. However, the authors annotated 12 differentially expressed protein spots with at least a two-fold difference in intensity (spots A1–A12; Figure 5). Among these 12 spots, six with the highest changes (spots A7–A12) were selected for MALDI-TOF MS analysis and identification (Figure 6). Based on PMF profile analysis, the six protein spots represented two proteins. Spots A7, A8, and A9 were isoforms of one protein, and spots A10, A11, and A12 were isoforms of another protein. The two proteins were successfully identified through PMF analysis. PMFs of the protein spots were used to search against the Swiss-Prot database (available online: https://www.uniprot.org/)using the MASCOT (Protein identification software for mass spectrometry data) search engine. Spots A7, A8, and A9 were identified as arginine kinase (accession number P51544), and spots A10, A11, and A12 were identified as fructose-bisphosphate aldolase (accession number AFX62893.1) (Figure 6). Whether these two differentially expressed proteins are biomarkers that could be used for the authentication of abalone from whelk requires validation through further experiments. However, the authors demonstrated that using TRIzol-prepared 2-DE as the first step to screen potential biomarkers is highly feasible. Moreover, this method provides a foundation for proteome study of highly valued dried food products and for the screening of potential biomarkers that could become ideal targets for the development of rapid, sensitive, convenient, and reliable immunobiosensors in the future.

## 3. Materials and Methods

### 3.1. Dried Seafood Products and Chinese Tonic Foods

Two dried seafood products, namely abalone slices (*Haliotidae* spp.) and whelk slices (*Buccinum* spp.), and two traditional Chinese tonic foods, namely caterpillar fungus (*Cordyceps* spp.) and lingzhi mushroom (*Ganoderma* spp.) were purchased from a local market. All samples were crushed and ground into fine powders. Sample powders (50 mg) were aliquoted into 1.5-mL Eppendorf tubes and stored at −80 °C for further use.

### 3.2. Trichloroacetic Acid (TCA)-Acetone Protein Precipitation Method

Protein was extracted from 50-mg seafood and traditional Chinese tonic food samples (powder) using 10% (*w*/*v*) TCA-acetone precipitation as described previously [24]. Briefly, a sample was added to 0.5 mL of lysis buffer (7 M urea, 2 M thiourea, 4% CHAPS, 40 mM Tris, pH 8.7) and subsequently lysed by sonication (5 min) on ice (Model cv18, SONICS). The lysed sample was then centrifuged at 14,000× *g* for 15 min at 4 °C to collect the supernatant. Subsequently, 2.5 mL of ice-cold 10% TCA-acetone was added to 0.5 mL of the protein extracts and maintained at −20 °C overnight for protein precipitation. Precipitated proteins were washed twice with ice-cold acetone. Finally, 0.5 mL of fresh lysis buffer was added to solubilise the protein pellet. The sample was then stored at −20 °C until further use.

### 3.3. TRIzol Protein Extraction Method

TRIzol reagent (1 mL; Roche, Basel, Switzerland) was added to 50 mg of powdered sample, and sonication for 5 min on ice as previously described [24]. Subsequently, 0.2 mL of chloroform was added to the suspension, which was then shaken for 15 s. The mixture was centrifuged at 14,000× *g* for 15 min at 4 °C after incubation at room temperature for 5 min to allow phase separation. The top layer containing RNA was completely removed. The lower layer of phenol was mixed with 0.3 mL of ethanol for DNA precipitation. The mixture was centrifuged at 2000× *g* for 5 min at 4 °C. The supernatant was then transferred to a new 2-mL Eppendorf tube, and approximately 1.7 mL of isopropanol was added. The mixture was incubated at room temperature for at least 20 min to allow protein precipitation. The protein pellet was collected by centrifuging the mixture at 14,000× *g* for 15 min at 4 °C. Subsequently, the protein pellet was washed with 95% ethanol before air drying. Finally, 0.5 mL of lysis buffer was added to solubilise the protein pellet, and the solution was stored at −20 °C until further use.

### 3.4. Protein Quantification

Protein quantification for urea-containing protein samples was performed using the modified Bradford assay (Bio-Rad, Hercules, CA, USA) [45]. Briefly, the extracted protein in 10 μL of lysis buffer was added into 240 μL of distilled water and mixed with 50 μL of Bradford reagent. Following incubation for 15 min, quantitation of the protein was performed using the absorbance read at 595 nm.

### 3.5. Two-Dimensional Polyacrylamide Gel Electrophoresis

Two-dimensional polyacrylamide gel electrophoresis was performed using the method reported previously [24]. Briefly, an 18-cm immobilised pH gradient (IPG) strip with a pH range of 4–7 (Bio-Rad, USA) was rehydrated with 340 μL of rehydration buffer (7 M urea, 2 M thiourea, 4% CHAPS, 0.2% dithiothreitol (DDT), 1% (*v*/*v*) IPG buffer, pH 4–7) for 16 h. The cup loading method was used. Following IPG strip rehydration, 100 μg of protein samples were added into the sample cup. IEF was performed using a Protean-IEF Cell (Bio-Rad, USA) and was conducted under the following conditions: 1 h at 100 V, 2 h at 300 V, 2 h at 1000 V, 2 h at 4000 V, and 5 h at 8000 V. Subsequently, the IPG strip was balanced with an equilibration buffer (50 mM Tris, pH 8.8, 6 M urea, 30% glycerol, 2% SDS, 1% DDT, a trace amount of bromophenol blue) for 15 min and, then, the IPG strip was placed in a fresh equilibration buffer containing 1% iodoacetamide (IAA) for another 15 min. The second dimension used 10% sodium dodecyl sulphate–polyacrylamide gel electrophoresis (SDS-PAGE). A constant current of 30 mA was applied to each gel until the bromophenol blue dye approached the bottom of the gel. Finally, silver staining was performed. The stained two-dimensional gel electrophoresis (2-DE) gel was scanned and saved in TIFF format. The images were analysed using Melanie VII (GeneBio, Genève, Switzerland). All protein samples were tested in triplicate, and the representative gel was shown.

### 3.6. In-Gel Protein Digestion and MALDI-TOF/TOF Analysis

Gel spots representing the proteins of interest were excised from the stained gel for in-gel tryptic digestion before MALDI-TOF MS analysis. The gel plugs were washed with 25 mM NH_4_HCO_3_ in 50% acetonitrile (ACN) three times, and the gel plugs were dehydrated with 100% ACN for 15 min. The gel pieces were reduced and alkylated through incubation with 10 mM DDT (55 °C for 45 min) and 55 mM IAA (dark conditions at room temperature for 30 min), respectively. In-gel tryptic digestion was performed by adding 20 ng/mL trypsin to 25 mM NH_4_HCO_3_ and leaving it overnight at 37 °C. Digested peptides were eluted with 0.1% trifluoroacetic acid (TFA) in 50% ACN. To perform MALDI-TOF MS analysis, 1 μL of the eluted peptide sample was mixed with 0.5 μL of saturated HCCA matrix solution (2 mg/mL of α-cyano-4-hydroxycinnamic acid in 0.1% TFA with ACN (2:1)) on an anchor-chip and allowed to dry. The dried mixture was washed with 0.1% TFA and subsequently recrystallised with a 1-μL mixture of ethanol, acetone, and 0.1% TFA at a volume of ratio 6:3:1. Samples were analysed through MALDI-TOF/TOF MS (Autoflex III; Bruker, Billerica, MA, USA) in reflection mode over a mass range of 700–3000 Da after calibrating with a peptide calibration standard (Bruker, USA). PMFs were generated from the combined spectra of 500 shots at different positions on the anchor-chip. Individual peptide ions in the PMF spectra were fragmented with collision-induced. dissociation (CID) to generate the MS/MS spectrum. Each spectrum was searched against the Swiss-Prot and National Center for Biotechnology Information (NCBI) non-redundant databases using the MASCOT search engine.

## 4. Conclusions

The generation of high-quality two-dimensional gel electrophoresis (2-DE) profiles is important for gel-based proteomic analysis. The authors evaluated the use of TRIzol protein extraction to produce 2-DE from two dried seafood products and two traditional Chinese tonic foods. Although lower protein yields were observed in the TRIzol extraction of abalone and whelk samples, high-quality 2-DE gels were obtained for all four samples through the TRIzol method, and the quality was comparable to that of the 2-DE gels generated through TCA-acetone precipitation in terms of numbers of spots, background signal, and resolution. TRIzol-prepared gels showed clearer backgrounds, higher intensity, and more spots than did the gels prepared using TCA-acetone in specific areas. The feasibility of identifying differentially expressed proteins from the 2-DE comparison of abalone and whelk samples was also demonstrated. This study established that TRIzol-based protein extraction provides simple, fast, reliable, and feasible sample preparation using a 2-DE–MS workflow for the proteomic analysis of dried seafood products and traditional Chinese tonic foods.

## Figures and Tables

**Table 1:** Samples prepared by using TRIzol-based extraction method for high-quality two-dimensional gel electrophoresis (2-DE).

Study Organism/Sample Type	Selected Sample(s)	Year	Reference
Halophile microorganisms	*Haloferax volcanii*	2006	[19]
Rat	Spinal cords segment	2007	[20]
Cell lines	Neck squamous cell carcinoma cell lines (SCC-25; FaDu)	2007	[22]
	MCF-7 cells	2009	[21]
	Human neuroblastoma cells (SH-SY5Y cells)	2012	[23]
Dinoflagellates	*Alexandrium* sp.; *Scrippsiella* sp.	2008	[24]
	*Alexandrium* *catenella*	2011	[27]
	*Alexandriumcatenella* (DH01)	2012	[25]
	*Lingulodinium* sp.	2012	[26]
	*Prorocentrum donghaiense*	2013	[28]
	*Alexandrium minutum* (AMTK4; AL1TAB); *Alexandrium tamarense* (ATCI01; SP3B8-3); *Gymnodinium catenatum* (GCHK; GCEO)	2015	[38]
Mites	*Dermatophagoides pteronyssinus*	2009	[29]
Plants	*Medicago truncatula* tissues	2011	[30]
Clinical samples	Human heart tissue	2008	[39]
	Caudal gland	2012	[31]
	Non-cancerous liver tissues	2013	[33]
	Heart biopsies	2015	[32]
Marine animals	*Mytilus galloprovincialis* (mussel); *Paralichthys olivaceus* (flounder); *Nereis diversicolor* (Polychaete)	2013	[36]
	*Haliotis diversicolor reeve*	2013	[34]
	*Haliotis gigantean reeve*; *Haliotis discus hannai lno*	2015	[35]
Reef corals	*Acropora hyacinthus*; *Acropora humilis*; *Acropora muricata*	2018	[37]
Dried seafood and dried tonic foods	Dried Abalone slices; Dried Whelk slices; Dried Ganoderma; Dried Caterpillar fungus	2018	This study

## Abbreviations

2-DE	Two Dimensional Gel Electrophoresis
AC	Acetonitrile
CHAPS	3-[(3-cholamidopropyl)dimethylammonio]-1-propanesulfonate
DDT	Dithiothreitol
HCCA	α-cyano-4-hydroxycinnamic Acid
IAA	Iodoacetamine
IEF	Isoelectric Focusing
MALDI-TOF	Matrix-Assisted Laser Desorption/Ionization Mass Spectrometry
PMF	Peptide Mass Fingerprint
SDS-PAGE	Sodium Dodecyl Sulfate Polyacrylamide Gel Electrophoresis
TCA	Trichloroacetic Acid
TFA	Trifluoroacetic Acid
